# Link between Flavor Perception and Volatile Compound Composition of Dark Chocolates Derived from Trinitario Cocoa Beans from Dominican Republic

**DOI:** 10.3390/molecules28093805

**Published:** 2023-04-28

**Authors:** Santiago Guzmán Penella, Renaud Boulanger, Isabelle Maraval, Gabi Kopp, Marcello Corno, Bénédicte Fontez, Angélique Fontana

**Affiliations:** 1 CIRAD, UMR Qualisud, F-34398 Montpellier, France; 2 Qualisud, Univ Montpellier, Avignon Université, CIRAD, Institut Agro, IRD, Université de La Réunion, 97490 Montpellier, France; 3 Barry Callebaut AG, Hardturmstrasse 181, 8005 Zurich, Switzerland; 4 MISTEA, Université Montpellier, INRAE, Institut Agro, 34060 Montpellier, France

**Keywords:** cocoa, chocolate, flavor perception, volatile compounds, flavor predictive models, PLS

## Abstract

The chemical composition of dark chocolate has a significant impact on its complex flavor profile. This study aims to investigate the relationship between the volatile chemical composition and perceived flavor of 54 dark chocolate samples made from Trinitario cocoa beans from the Dominican Republic. The samples were evaluated by a trained panel and analyzed using gas chromatography-mass spectrometry (GC-MS) to identify and quantify the volatile compounds. Predictive models based on a partial least squares regression (PLS) allowed the identification of key compounds for predicting individual sensory attributes. The models were most successful in classifying samples based on the intensity of bitterness and astringency, even though these attributes are mostly linked to non-volatile compounds. Acetaldehyde, dimethyl sulfide, and 2,3-butanediol were found to be key predictors for various sensory attributes, while propylene glycol diacetate was identified as a possible marker for red fruit aroma. The study highlights the potential of using volatile compounds to accurately predict chocolate flavor potential.

## 1. Introduction

Chocolate is highly consumed in developed markets and is increasingly penetrating new markets, mainly in Asia and Latin America. In 2021, the global chocolate market was worth USD 46.6 billion and is expected to grow at a compound annual growth rate of 4.98% from 2022 to 2028, expecting to be valued at USD 65.49 billion by the end of this forecast period [1].

While 90% of the world’s total “fine” cocoa exports come from Latin America, the Dominican Republic is one of the three biggest exporting countries, along with Ecuador and Peru. In broad terms, fine “cocoa” is mostly produced from the Criollo or Trinitario varieties and is distinguished from “bulk” cocoa because of flavor alone. Fine cocoa often exhibits fruity, floral, herbal, woody nutty, and caramel-like notes [2].

Cocoa products from Dominican Republic beans are known to display multiple dominant flavor profiles, ranging from low cocoa and bitter, to winey, earthy, and spicy notes [3]. Furthermore, Trinitario cocoa has been traditionally known as fine-grade cocoa [4], and has been associated to varied sensory attributes, including a marked chocolate flavor and distinctive wine-like notes [5].

Flavor is one of the most significant consumer acceptance parameters. It is hence one of the main quality indicators for cocoa products. Cocoa flavor is highly complex as many diverse components may be linked to its resulting sensory perception. Both non-volatile and volatile compounds contribute to the overall flavor profile of cocoa and cocoa-derived products. Over 600 odor compounds have been reported to be found in cocoa and chocolate [6].

The aroma composition of cocoa products is tightly related to the unique postharvest processing conditions, as well as to the variety and the origin of the cocoa itself [7].

Among the non-volatile compounds in cocoa, alkaloids and polyphenols have arguably the highest impact on flavor perception, as they are both linked to bitterness. Additionally, polyphenols are associated to astringent sensations and contribute to green and fruity flavors [8]. Moreover, proteins and carbohydrates are non-volatile compounds that play an important role in the formation of volatile aroma compounds during the drying, roasting and conching processes by means of Strecker Degradation and a Maillard reaction [6].

On the other hand, volatile aroma compounds found in cocoa products include esters, alcohols, acids, and phenols, which are mostly derived from the fermentation and drying processes. These compounds tend to be linked to sweet, sour, fruity, and floral notes, with the exception of phenols, which may convey smoky and other generally undesirable hints [9]. Pyrazines, aldehydes, and ketones products resulting from Maillard reactions are other volatile compounds of interest. Some ketones and aldehydes are known to bestow floral notes to cocoa, but pyrazines are usually associated to the expression of nutty, earthy, roasted, and green notes [10].

The use of gas chromatography-mass spectrometry (GC-MS) for the characterization of the volatile composition of chocolate and other cocoa products is a well-established technique [1,5].

In recent years, multiple studies have been conducted in an attempt to better understand the impact of different variables, such as the plant genetic origin or processing conditions, on the volatile composition and on the flavor perception of cocoa and chocolate [11,12,13,14,15,16,17,18,19,20]. However, these studies did not always look for the relationship existing between the volatile composition and sensory perception in chocolate. Many of them have tried to identify compounds that could be used as markers for certain flavor attributes [21,22,23,24,25]. While some have looked into the single compound’s presumed impact on the actual perceived taste of chocolate itself [26,27,28,29], most have focused solely on the individual compound’s reported sensory descriptor, independent to the food matrix itself. Additionally, some studies have either contradicted or have not been able to corroborate previously reported results when trying to establish the individual contribution of single compounds to the overall perceived flavor profile in final products, as has for instance been the case of linalool’s impact on floral notes [30]. A rather limited number of studies have indeed attempted to describe and predict the perceived intensity of sensory attributes based solely on the volatile composition of the cocoa products [31,32,33]. Some others have classified samples into differentiated sensory groups based on their overall volatile composition and sensory profile, but without distinguishing between unique sensory attributes [34]. A 2022 study claimed to be the first to show how distinct differences in the flavor profiles of dark chocolates are reflected in their molecular compositions, while also factoring in the compounds’ odor activity values (OAC) and dose over threshold factors (DoT) [30].

In spite of the growing interest in researching both volatile composition and flavor in cocoa and chocolate, identifying the linkage between the two remains particularly challenging because of the established high chemical complexity of the product. Moreover, the differences among chocolates produced from beans of the same variety and origin are bound to be more nuanced compared to those found in chocolates derived from cocoa that have more obviously differentiated backgrounds. Furthermore, the ability to differentiate chocolates in terms of their flavor profile in a fast, repeatable, and reliable way is of major interest for the industry, notably in the case of highly-sought-after attributes, such as fruity or floral.

The focus of this study is to identify key volatile aroma compounds that may enable differentiation within a selection of dark chocolate samples derived from Trinitario beans grown in the Dominican Republic, as well as to ultimately develop sufficiently accurate predictive models that would allow the discrimination of samples with regard to their flavor potential. This characterization will be based on the perceived sensory profile of each of the samples, blind to their processing conditions, with the aim of understanding to what extent subtle differences in taste could be described by differences in volatile composition.

## 2. Results

### 2.1. Sensory Characterization of Dark Chocolate Samples

**Figure 1:**
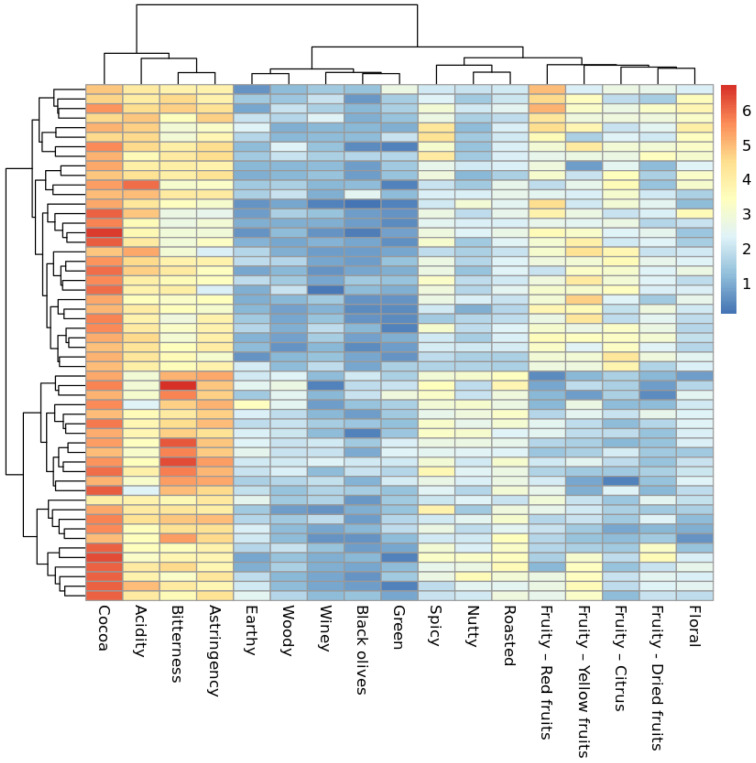
Clustered heatmap of all samples (rows) in relation to their mean sensory scores per attribute (columns). The score scale ranges from 1 to 10; the highest granted mean sensory score was 6.

A clustered heatmap (Figure 1) allows for a summarized visualization of the raw (not pretreated data, as in not centered nor scaled) *mean sensory scores* of all 54 chocolate samples evaluated in this study, along with insights into the correlations that exist among the different attributes that were evaluated (sweetness, bitterness, acidity, astringency, cocoa, yellow fruits, red fruits, citrus, dried fruits, nutty, winey, black olives, green, earthy, floral, woody, spicy, and roasted). In Appendix A, Table A1, additional information on the overall sensory results obtained from the chocolate dataset is presented.

It can be seen that cocoa, acidity, bitterness, and astringency are attributes highly correlated with each other. Moreover, cocoa is the attribute that displays the highest overall *mean sensory scores*, which would indicate that the majority of the chocolate samples display a flavor profile that has strong cocoa notes.

All of the fruity sub-attributes (red fruits, yellow fruits, citrus, and dried fruits), together with the floral attribute, also present a high degree of correlation based on their *mean sensory scores*. These fruity *mean sensory scores* show their highest values mostly whenever the values for nutty, roasted, and spicy attributes are the lowest.

The earthy, woody, winey, and black olives attributes present the lowest *mean sensory scores* among all attributes. The flavor profiles of the set of chocolate samples evaluated are thus characterized for weakly expressing these attributes. Furthermore, earthy, woody, winey, and black olives seem to show their highest *mean sensory score* values whenever the values for bitterness and astringent are also high.

A principal component analysis (PCA) was performed on the *mean sensory scores* of all sensory attributes obtained for all samples. This was performed to characterize how the differences in the reported *mean sensory scores* were driving sample differentiation with regards to their sensory profiles. The two main components, F1 and F2, were able to explain 51.86% of the variance among samples.

**Figure 2:**
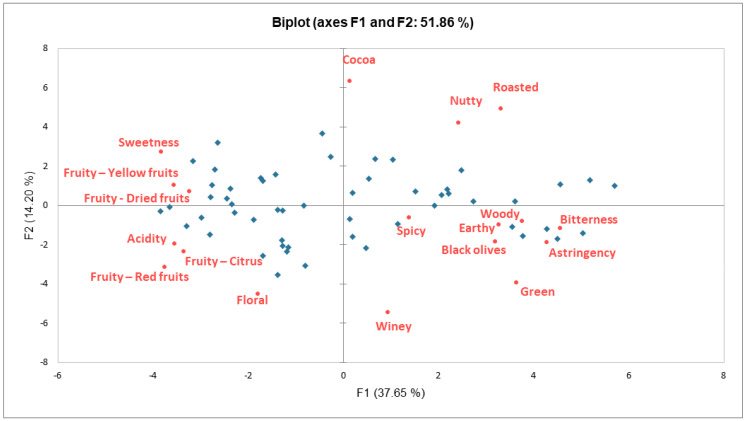
F1−F2 biplot of PCA on mean sensory scores showing distribution of samples (observations, in blue), and of sensory attributes (variables, in red).

**Figure A1:**
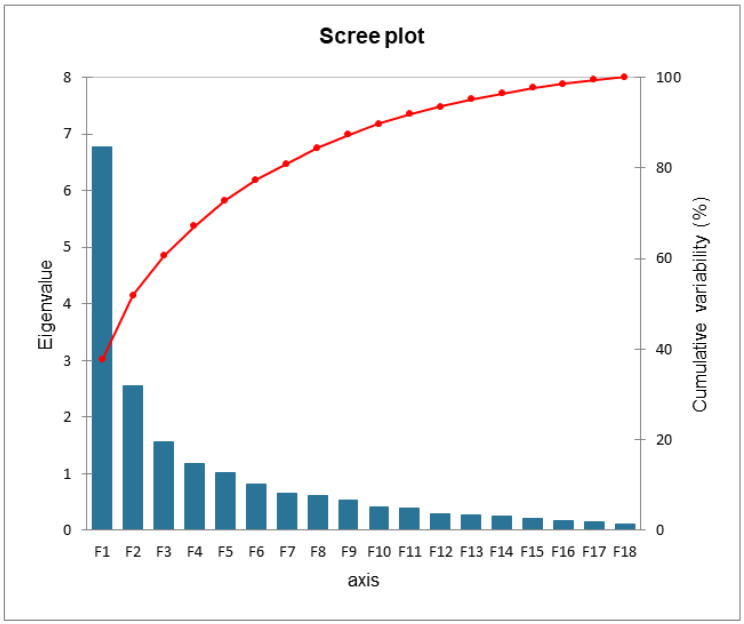
Scree plot of the PCA performed on the mean sensory scores obtained for all sensory attributes; linked to Figure 2.

In Figure 2, the PCA biplot showing the distribution of all chocolate samples in the space of F1 and F2 is presented, together with the loadings, corresponding to all sensory attributes (variables). The scree plot pertaining to this analysis is shown in Appendix A, Figure A1. It indicates that the first two components are responsible for a substantial amount of the variability in the data, while the third component explains a relatively small amount of variance.

The samples seem to be well scattered along both axes. The distribution along the F2 axis is presumably defined by the direction of cocoa and winey attributes. Furthermore, groups of samples can be observed in the whole F1-F2 plane, which could be potentially described in terms of the membership to each of the different quarters.

The samples exhibiting high roasted and nutty *mean sensory scores* are opposed to samples with high *mean sensory scores* for acid, floral, and citrus attributes. Similarly, samples whose *mean sensory scores* are high for sweetness, yellow fruits, and dried fruits, are opposed to samples high in woody, spicy, earthy, black olives, green, bitter, and astringent mean sensory scores.

### 2.2. Identification and Quantification of Volatile Aroma Compounds Present in the Dark Chocolate Samples

Volatile compounds present in the 54 dark chocolate samples were determined by GC-MS; 34 known compounds were identified to be present from well-defined peaks shown in the retrieved spectra. An unidentified 35th compound was found in several of the samples, but it could not be associated to any of the spectra already in the NIST Mass Spectral Library. All identified volatiles, together with the odor descriptors commonly associated individually to each, are listed in Table 1.

In Appendix A, Table A2, additional information is presented on the relative concentrations obtained for each of the identified volatile compounds among the chocolate sample dataset.

**Figure 3:**
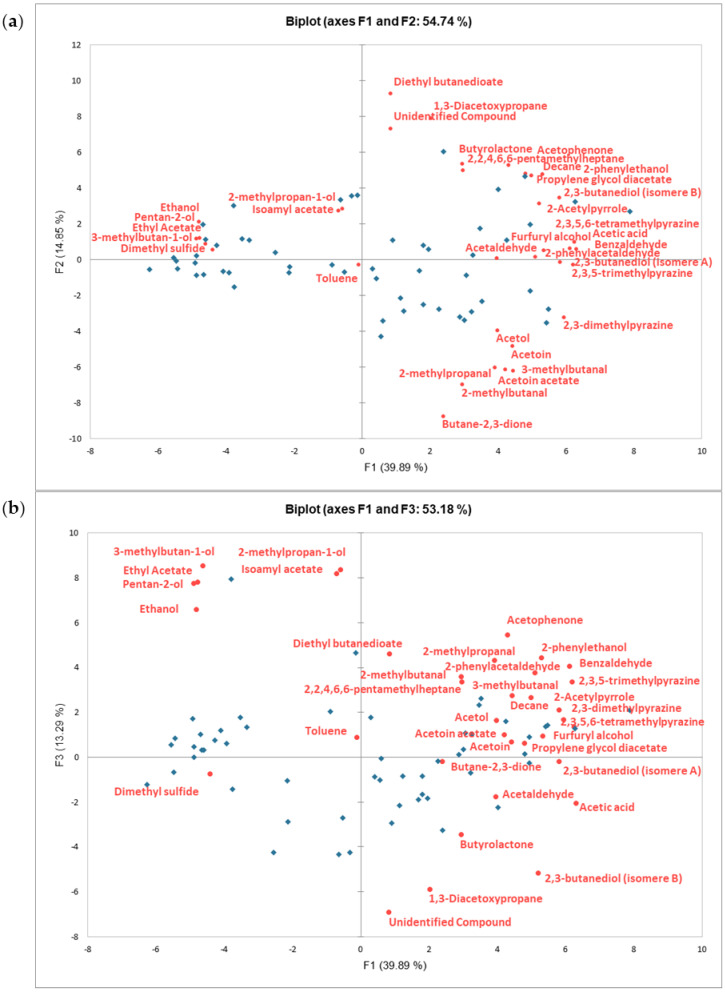
Biplots of the PCA on the relative concentration of the identified volatile compounds, showing distribution of samples (observations), and of volatile compounds (variables): (**a**) F1−F2 biplot; (**b**) F1−F3 biplot.

**Figure A2:**
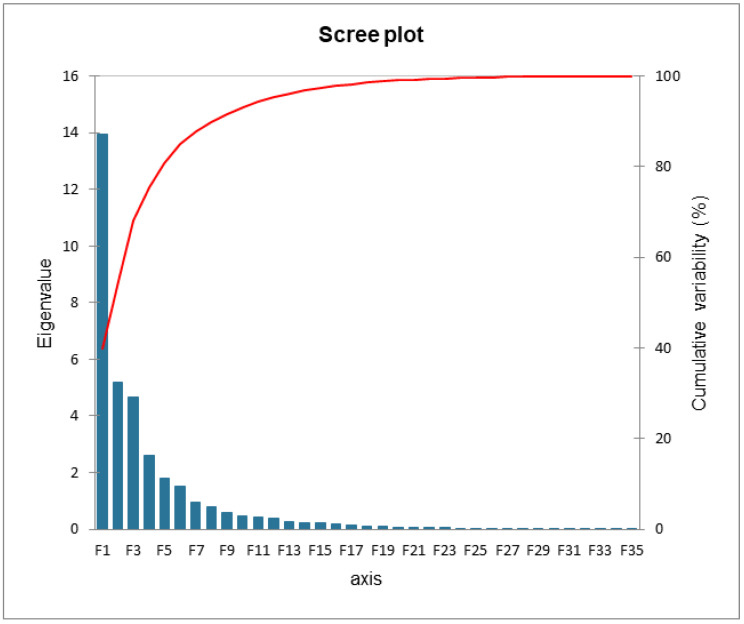
Scree plot of the PCA performed on the relative concentrations of the identified volatile compounds; linked to Figure 3.

A PCA was performed on the relative concentrations of the identified volatile compounds. While the two main components (F1 and F2) were able to explain 54.74% of the variance among the samples, the contribution of the third component to the explanation of the variance of the dataset is also worth discussing. This is because the third component appears to explain a sufficiently large amount of variance (as seen in the scree plot pertaining to this PCA, shown in Appendix A, Figure A2). Together, F1 and F3 are capable of explaining up to 53.18% of the dataset variance. In Figure 3, the PCA biplot showing the distribution of the chocolate samples in the space of F1 and F2, as well as in the space of F1 and F3, is presented, together with the variable loadings, corresponding to all volatile compounds.

Within the space of the first two components, the samples are well scattered. A segregation of the samples is predominantly observed in a gradient along the F1 axis, pointing towards a somewhat well-defined formation of two distinct groups. F1 sample differentiation appears to be mainly driven by the relative concentrations of pentan-2-ol (whose contribution to the building of the component is of 3.46%), ethanol (3.36%), ethyl acetate (3.32%), 3-methylbutan-1-ol (3.09%), and dimethyl sulfide (2.83%) on one side. While on the opposite extreme of the F1 axis, the most contributing volatile compounds leading the differentiation of the samples are acetic acid (contributing to the component at 5.79%), 2,3,5,6-tetramethylpyrazine (5.70%), 2,3,5-trimethylpyrazine (5.60%), benzaldehyde (5.45%), 2,3-dimethylpyrazine (5.15%), and 2,3-butanediol (isomere A; 4.93%).

Differentiation of the samples along the F2 axis is less pronounced, compared to what was seen along the F1 axis. No evident grouping of samples is seen here and the gradient segregation is not as widely dispersed. Nonetheless, the volatile compounds contributing the most to this second component may be clearly identified as diethyl butanedioate (12.54%), 1,3-diacetoxypropane (9.10%), and the “unidentified compound” (7.81%) on one side. The following compounds may be found towards the opposite direction, along the components: axis butane-2,3-dione (11.13%), 2-methylbutanal (7.10%), 3-methylbutanal (5.63%), acetoin acetate (5.50%), and 2-methylpropanal (5.31%).

It may then be assumed that differences in the relative concentrations of the above-listed compounds for both F1 and F2 are contributing the most to the differentiation of samples, in relation to their overall volatile composition.

Furthermore, it has been observed that sample segregation is not necessarily associated with differences in the relative concentrations of entire chemical families, but rather appears to be linked to specific, unique compounds that are independent of their chemical family affiliation. This observation suggests that the sensory attributes of samples may be more closely associated with individual compounds, rather than the chemical families to which they belong.

In relation to the biplot on the F1 and F3 components, it must be noted that the dimension of the third component appears to be mostly represented by one single chocolate sample (contributing to 25.13% of the building of the component), which would indicate this sample is an atypical individual. This atypical sample seems to be characterized by compounds such as 3-methylbutan-1-ol (which is contributing to 10.55% of the third component), 2-methylpropan-1-ol (10.18%), isoamyl acetate (9.73%), ethyl acetate (8.88%), pentan-2-ol (8.75%), and ethanol (6.27%). It is only when looking at the third dimension that these compounds are visually separated along its axis from dimethyl sulfide. Other compounds whose distribution in relation to the other compounds changes markedly along the axis of the third component are 1,3-diacetoxypropane and the unidentified compound, both of which are also important contributors to the building of these components, and which now seem to be clearly distanced from diethyl butanedioate, for instance.

### 2.3. Identification of Key Aroma Compounds Based on Their Impact on the Sensory Perception of Dark Chocolate Samples

**Figure 4:**
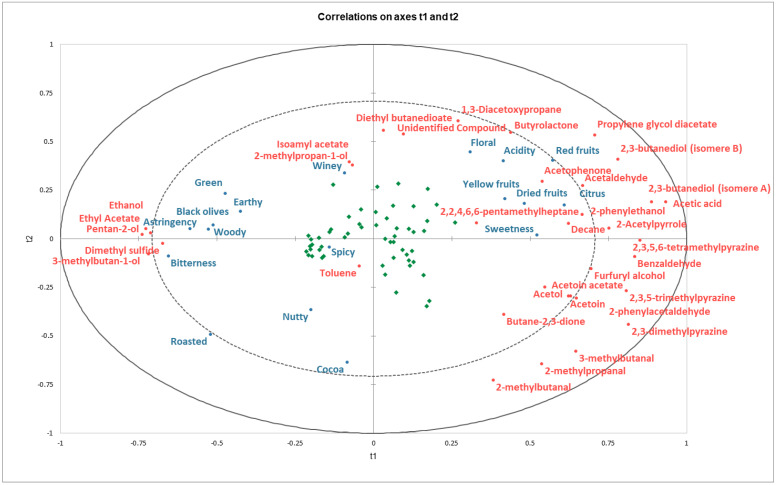
Correlation map generated by PLS regression between the components, explanatory variables, and response variables, along the t1 and t2 axes, with the chocolate samples superimposed.

**Figure A3:**
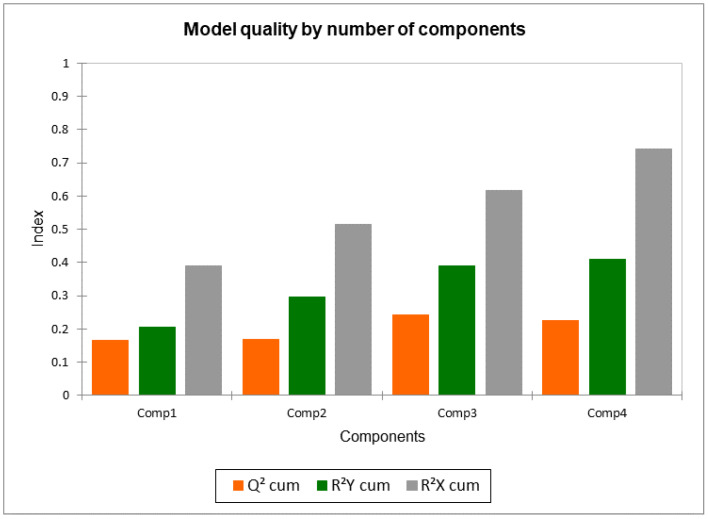
Bar chart of the quality of the PLS regression as a function of the number of components. Q^2^ cumulated index stands for the global goodness of fit; R^2^Y and R^2^X cum stand for the correlations existing between the components and the explanatory (X, relative concentrations of volatile compounds) and dependent (Y, mean sensory scores) variables.

A partial least squares (PLS) regression was performed in an attempt to obtain a global view of the whole dataset. This allows the principal relationships existing between groups of volatile compounds (explanatory variables) whose concentrations may be able to predict *mean sensory scores* (response variables) to be summarized. The variables were centered and reduced. The quality of the PLS model obtained is explained using a bar plot presented in Appendix A, Figure A3; it shows that while the first two components summarize the correlations between explanatory and dependent variables well, the third component may still provide additional information. The obtained bi-dimensional correlation plot on axes t1 and t2 is presented below, in Figure 4.

This overview of the PLS regression showed a number of similarities with the two PCAs that had been already performed on both *mean sensory scores* and on the relative concentration of the volatile compounds. Taken as a whole, this PLS regression confirmed that the samples are broadly differentiated in two groups. One of these groups could be described as highly aromatic for being linked to large concentrations of plenty of different volatile compounds, which at the same time seem to describe higher fruity, floral, and sweet *mean sensory scores*. On the other hand, a group of samples seems to be characterized by lower concentrations of most of the identified volatiles and by exhibiting higher *mean sensory scores* for astringent and bitter attributes, among others.

The PLS regression demonstrated that all fruity attributes remain seemingly very highly linked, just as it was seen with the PCA. Not only is the link between the different fruity attributes maintained, but the relationship existing between them and the acid, sweet, and floral attributes appears to be further tightened. Moreover, these attributes seem to be very tightly correlated to the differences in the relative concentration of numerous volatile compounds, including but not limited to acetic acid, 2,3-butanediol, propylene glycol diacetate, and 2,3-dimethylpyrazine.

Being located closer to the center of the plot, attributes such as spicy, winey, and nutty appear to be amongst the most difficult to predict. This could indicate that differences in their *mean sensory scores* are not well described by differences in the relative concentrations of the volatile compounds. Noticeably for spicy, nonetheless, it was already seen in the PCA that its contribution to the first two principal components was moderate and was thus not driving sample segregation as much as the other attributes were. This might suggest that the *mean sensory scores* for spicy, winey, and nutty are overall too low and similar for them to drive sample differentiation altogether (or that they could be better represented in the other PCA components).

The attributes of cocoa and roasted remain closely linked, as seen in the PCA, and also appear to be largely described by lower relative concentrations of most of the volatile compounds.

Similarly, the PLS regression suggests that the attributes green, earthy, black olives, woody, bitterness, and astringency are as related as initially observed in the PCA. It would also seem that they may all be well characterized by higher relative concentrations of ethanol, ethyl acetate, pental-2-ol, dimethyl sulfide, and 3-methylbutan-1-ol.

Lastly, there is a subgroup of samples that exhibit high relative concentrations of compounds, including but not restricted to 2,3-dimethylpyrazine, 3-methylbutanal, 2-methylpropanal, and 2-methylbutanal, but which are seemingly not well characterized by any single sensory attribute.

**Figure A4:**
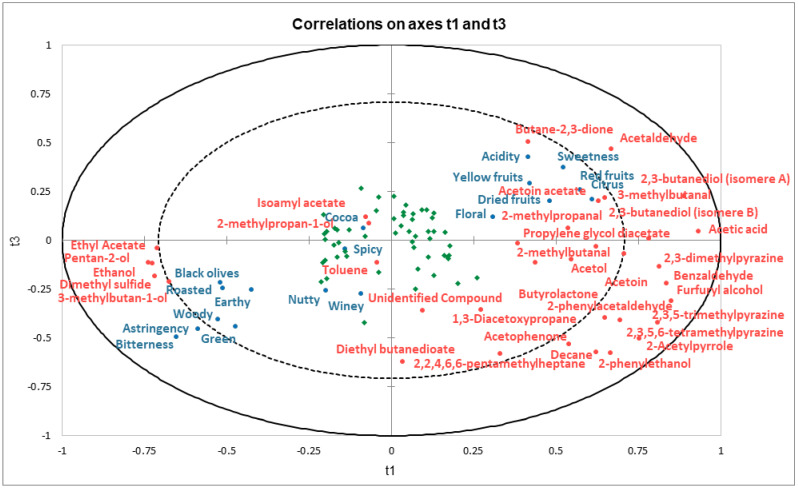
Correlation map generated by the PLS regression between the components, explanatory variables, and response variables, along the t1 and t3 axes, with the chocolate samples superimposed.

The bidimensional correlation plot on axes t1 and t3 is also presented as complementary information (Appendix A, Figure A4). It shows how compounds such as acetaldehyde and butane-2,3-dione are closely grouped together and relatively separated from the rest along the t3 axis, being now more closely related to sensory attributes such as acidity and sweetness. This might be giving hints at these compounds’ potential ambivalent character, being linked to multiple sensory attributes at once.

### 2.4. PLS Predictive Models for Individual Sensory Attributes

It would be of interest to gain an in-depth understanding of the specific compounds contributing to predicting each attribute. For this purpose, PLS predictive models were built. Given the results of the prior global PLS regression analysis, which already established some key relationships between the explanatory and response variables, it is not expected that a substantial difference will be observed.

For each attribute, the samples were classified in two groups relative to their *mean sensory scores*: samples above the median and samples below the median. This classification would enable samples to be broadly differentiated in terms of their flavor potential per attribute, delimited by the current sample set.

As described in Section 4. Materials and Methods, training models were built and optimized by cross-validation. Predictive models were then built for the best fitted training models. The model performance metrics for each of the obtained models are presented in Table 2.

The five volatile compounds that had the largest impact in the building of each model (most important variables) were retrieved for reporting. A Pearson correlation analysis was performed on the raw data in order to understand the nature of the correlations existing between the explanatory (volatile compounds) and the response variables (sensory attributes). Both the most important compounds per predictive model and their corresponding Pearson correlation coefficients (obtained from the raw data) are presented in Table 3.

Acetaldehyde seems to be a key compound in predicting multiple distinct models, and it is either positively or negatively related to an increase in the attribute’s intensity. Other compounds whose importance is prevalent in the building of several models are the two isomeres of 2,3-butanediol, as well as dimethyl sulfide, followed by 3-methylbutan-1-ol, acetic acid, 3-methylbutanal, pentan-2-ol, ethyl acetate, and butyrolactone. The rest of the listed compounds seem to be more attribute-dependent, as they present the highest importance in describing single unique attributes.

A different and more restrictive classification of the samples was then performed. The samples were classified as “high” based on their mean sensory scores, with the highest quartile of the scores being considered for the classification. Subsequently, the models were developed as previously described based on this newly established classification of the samples. ROC was again used as the metric. If was found that the accuracy, specificity, and sensibility of most models decreased considerably, except for those of bitterness and astringency. The best models obtained for both bitterness and astringency are presented in Table 4.

There is only one compound whose importance markedly differs when attempting to predict whether a sample is highly bitter as opposed to merely above the median, and this is 2,3-butanediol (isomere B), which is now listed among the top five most important variables. This would suggest that these compounds have a stronger differentiating power than the rest when attempting to discriminate the samples that express a higher intensity of bitterness.

While for astringency, on the other hand, there is a more pronounced rearrangement of the importance of the variables responsible for predicting if a sample will be classified as highly astringent, as acetic acid, 2,3-butanediol (isomere B) and 3-methylbutan-1-ol now acquire much more importance.

## 3. Discussion

Given that the set of chocolate samples evaluated in the context of this study were produced from cocoa of the same variety and region, their flavor profiles were not expected to be as different as if chocolates produced from different regions and genotypes had been included. Nonetheless, while most samples displayed a dominant cocoa profile, important differences were found in the expression of most other attributes. These differences translated into certain samples displaying markedly differentiated fruity notes as opposed to others exhibiting more of a bitter/astringent/spicy/winey profile, covering a wide flavor range within the varietal limitations of the cocoa in this region.

The grouping of the samples based on their sensory profiles (Figure 2) showed certain parallels to what has been reported in similar studies conducted on the organoleptic properties of cocoa products, where PCA plots have also grouped together attributes such as astringent, bitter, and green, opposed to fruity, floral acid, or cocoa notes, for instance [39].

All of the identified compounds had been previously reported in the literature as present either in dry fermented cocoa beans, roasted beans, liquor, or dark chocolate from different varieties and regions, processed under unique conditions [14,17,18,20,32,38,39,40,41,42,43,44,45,46]. Hence, none of the 35 identified compounds point to being unique markers that could be potentially used for differentiating the Dominican Republic chocolates that make up the sample dataset of this study in relation to other cocoa products.

The predictive models obtained from the PLS regression are limited in their predictive capacity due to the reduced number of samples used, resulting in a data-dependent model. Despite this limitation, the models still display a relatively good level of predictive accuracy. It is important to be cautious when interpreting the results of the analyses aimed at identifying the key compounds that could predict the perceived intensity of the evaluated sensory attributes. There are several confounding factors that must be taken into consideration, such as variable exchangeability and causality [47].

Simply identifying a compound as having an important relation with a particular attribute does not necessarily imply causality. To establish causality, it would be necessary to consider other factors that may be contributing to the expression of the sensory attribute and to compare with previously reported findings. Variable exchangeability refers to the presence of highly correlated variables, both explanatory and response variables, which can lead to misleading conclusions about the importance of a particular compound in predicting sensory attributes.

Both variable exchangeability and causality will now be further discussed in relation to the results obtained in an attempt to determine whether causality may be assumed. By carefully considering both of these factors, a deeper understanding of the mechanisms of sensory perception may be gained and more accurate predictions about the impact of individual compounds on the perception of sensory attributes may then be conducted.

When attempting to identify the most important compounds responsible for contributing to the perception of each individual sensory attribute, it was seen that the existing underlying correlation was often negative. This could indicate that the presence of certain volatiles was detrimental to the perception of the given attribute, or in other words, that low concentrations of said compounds were needed to maximize the intensity of the described attributes.

The above statement seems particularly true for bitterness and astringency. It is worth noting that most of the compounds identified as important for predicting both bitterness and astringency hold a negative correlation with the intensity of both attributes, which would mean that the presence of these compounds in low concentrations would be needed for a sample to be highly bitter and astringent.

Bitterness (classified as one of the four primary tastes, along with sweet, sour, and salty) and astringency (a trigeminal sensation) have both been strongly linked in cocoa products to mostly non-volatile compounds [48]. Theobromine and caffeine, for instance, are alkaloids that contribute to the typical bitter taste of cocoa [6]. Polyphenols and flavonoids such as tannins, flavan-3-ols [(+)-catechin, (-)-epicatechin and (-)-epigallocatechin], and anthocyanins have also been associated with an astringent and bitter taste [49].

Nonetheless, it would appear that the perception of both bitterness and astringency increases whenever the concentration of compounds known to be linked to unpleasant notes is relatively high, as it happens with dimethyl sulfide and 3-methylbutan-1-ol, while the perceived intensity seems to decrease whenever the sample contains higher concentrations of compounds linked to pleasant notes, such as the sweetness associated with 2,3-butanediol, the chocolate taste linked to 3-methylbutanal and 2-methylpropanal, the acidity of the acetic acid, and the fruity-like odor of the acetaldehyde. This could suggest that these desirable compounds may have a particularly important masking effect over the non-volatile-associated bitterness and astringency, as well as over the high concentrations of volatile compounds responsible for the unpleasant notes that may be reminiscent to the bitter taste and the astringent sensation.

In order to better understand the real impact of these compounds’ potential “masking effect” on the reduced perception of bitterness and astringency in chocolate, further analysis would be needed to also take into account the concentration of non-volatile bitter and astringent compounds and to look deeper into the existing correlations.

It is noteworthy that bitterness and astringency exhibit a relatively high degree of correlation, with a Pearson correlation coefficient of 0.732. This correlation is reflected in the shared compounds that were selected as important for predicting both sensory attributes (acetaldehyde, dimethyl sulfide, and 2,3-butanediol (isomere A)). This could also potentially mean that the volatile compounds identified as important in describing bitterness are also likely to be important in describing astringency. This suggests that compounds such as 2-methylpropanal and 3-methylbutanal, which are related to a chocolate-like flavor, may be important when describing bitterness, as well as compounds such as 3-methylbutan-1-ol, which has a pungent taste, in describing astringency. This highlights the potential for overlapping contributions of certain volatile compounds in both attributes, further emphasizing the interconnection of bitterness and astringency in the flavor profile of dark chocolate.

The acidity, which is another taste that was accurately described and predicted by the set of identified and quantified volatiles in this study’s samples, was unsurprisingly largely influenced by high concentrations of acetaldehyde, which has an acidic taste. Surprisingly, on the other hand, the differences in the concentration of acetic acid appeared not to have an important effect on the increased perception of acidity, which would have been expected.

Other volatiles found to be seemingly important in describing the acid taste were not necessarily acidic in nature (butyrolactone, isoamyl acetate, and 2,3-butanediol), leaning more towards sweet and fruity notes. This could suggest that such compounds may have a potential enhancing effect on the perception of the volatile acidity brought in by acidic volatile compounds such as acetaldehyde and acetic acid, as well as by the non-volatile acidity, for which phosphoric and lactic acids are mostly responsible, together with oxalic, malic, succinic, and citric acids [50].

Nonetheless, another possible explanation would be that some of these non-acidic compounds are actually statistically exchangeable with the acetic acid or other volatile compounds with an acid-like taste. When looking into the existing correlations between the relative concentrations of volatile compounds, it may be seen that the acetic acid is very highly correlated with most of the compounds here identified as important in describing acidity: 2,3-butanediol (isomere A; Pearson correlation coefficient of 0.673), 2,3-butanediol (isomere B; 0.850), acetaldehyde (0.673), and butyrolactone (0.595). This could then imply that some of these compounds were selected as important variables in the PLS regression because they were providing similar information compared to that of the acetic acid. This redundancy would make it difficult to determine which variable is the most important predictor and could not allow us to discard acetic acid as a likely important contributor in the expression of acidity.

Derived from alanine, acetaldehyde is one of the most abundant Strecker aldehydes linked to chocolate notes [51], and it is one of the most abundant carbonyl compounds found in many fermented foods [52]. Acetaldehyde was found to be of high importance in describing and predicting most of the sensory attributes discussed in this study. This compound is mostly formed during alcoholic fermentation, by the decarboxylation of pyruvate, after which it may subsequently be transformed into ethanol by alcohol dehydrogenase enzymes [53]. Acetaldehyde is also an intermediate in the synthesis of acetic acid and acetoin, which may later be reduced to 2,3-butanediol [51]. While acetaldehyde’s associated flavor is commonly described as “oxidized”, studies conducted into its impact on the flavor perception of wines have shown that, at different concentrations, it may be linked to markedly differentiated sensory notes, ranging from fresh fruit aromas at low concentrations, to nutty, cocoa, ripe fruit, and even rotten-like off-flavors at higher concentrations [53].

The numerous aroma descriptors that have been associated to different concentrations of acetaldehyde could help to explain why it has also been identified as a key compound when attempting to explain different chocolate descriptors in this present study. Additionally, it must also be considered that interactions of acetaldehyde with other molecules may not only affect its flavor perception in chocolate, but may also alter the perception threshold of free acetaldehyde [53].

In addition, it has been noted that during cocoa fermentation, acetaldehyde reacts with epicatechin and procyanidin B2 to form ethyl-linked flavan-3-ol trimers [54]. This could be the reason behind the observed negative correlation between the relative concentration of acetaldehyde and the bitterness *mean sensory scores*, as it would suggest that a greater amount of the available acetaldehyde was involved in the formation of non-volatile bitter compounds through condensation reactions.

The statements above provide a reasonable explanation for the causal relationship between the increased acetaldehyde content and the heightened perception of certain attributes such as fruitiness, as well as the reduced perception of bitterness and astringency. Furthermore, these deduced causal relationships are reinforced by the lack of strong correlation between the relative concentration of acetaldehyde and that of any other compound, which reduces the likelihood of mistakenly interpreting the impact of this compound as it would not be easily exchangeable.

Similarly, while dimethyl sulfide (a sulfur compound widely present in food products) on its own has been described as exhibiting green and unpleasant cabbage-like aromas [55], studies about its impact on other aroma descriptors in wine as a matrix have been published. It has been suggested that dimethyl sulfide may play a role as a fruity flavor enhancer, especially in the case of blackberry and blackcurrant aromas [55]. Furthermore, the presence of dimethyl sulfide has also been linked to a decrease of the olfactory threshold of fruity notes and to an increase in overall flavor intensity. At higher concentrations, on the other hand, it has been reported as displaying notes more resembling black olives and truffles [55]. The results obtained seem to point to a similar direction in chocolate. Based on the retrieved Pearson correlation coefficients, it would seem that higher concentrations of this compound are presumably responsible for enhancing the expression of bitterness and astringency, as well as the perceived green and black olive aromas. It would also seem that its presence in low concentrations could indeed play an important role in enhancing a red fruit aroma and sweet taste in the dark chocolate samples evaluated.

Dimethyl sulfide seems to exhibit no strong correlation with any other compound, indicating that no other volatile compound is providing similar information as dimethyl sulfide. This suggests that the conclusions drawn about it are likely valid.

Another compound whose importance seems to be prevalent in describing and predicting several of the studied sensory attributes is 2,3-butanediol. The present study’s results show that higher concentrations of this compound appear to be related to a higher overall flavor intensity, particularly in the case of desirable notes such as citrus, red fruits, and sweetness; whereas lower concentrations seem to be linked to more intense bitterness and astringency, which are attributes that tend to be associated with lower quality chocolate. The obtained results would then be in agreement with previously reported propositions that suggest that the presence of this compound is desirable for high quality cocoa products [56].

As expected, the two identified isomers of 2,3-butanediol show a strong correlation with each other, suggesting that the information they are each providing is redundant when predicting any of the given attributes. Due to this exchangeability, the correlation they have with other compounds is also quite similar, as is the case with their correlation with acetic acid.

The identification of propylene glycol diacetate as a key predictor is noteworthy. This volatile compound has been described as having a fruity aroma [43] and it has now been found to be an important variable when determining the potential intensity of a red fruit aroma in chocolate samples. Its high correlation with the red fruit sensory attribute and its unique importance in predicting the expression of red fruits make it a valuable marker in this regard. It is the second most important predictor of red fruit expression after acetaldehyde, suggesting that there may be a combined effect on this particular fruity attribute. Additionally, propylene glycol diacetate does not appear to be strongly correlated to any compound other than 2,3,5,6-tetramethylpyrazine and acetophenone, having Pearson correlation coefficients of 0.739 and 0.733, respectively. Since neither of these two compounds are linked to expressing red fruit-related aromas, it may then be assumed that the information provided by them is not similar and that propylene glycol diacetate might indeed be responsible for the expression and perception of a red fruit aroma in the chocolate samples evaluated.

## 4. Materials and Methods

**Dark Chocolate Samples**—Barry Callebaut AG provided 54 different dark chocolate samples, which were produced following internal and non-disclosed processing parameters. All chocolates were produced from cocoa beans issued from a fermentation campaign of Trinitario beans carried out by Barry Callebaut AG in the Dominican Republic, in April 2020. The recipe of the chocolates was described as containing: 60% cocoa liquor, 30% sugar, and 10% deodorized cocoa butter; no lecithin was added. The tempered chocolates were molded in plastic molds into individual chocolate square-shaped pieces (3.5 cm × 3.5 cm), each weighing approximately 5 g. The molded chocolates were vacuum sealed and allowed to stabilize for a month at room temperature. The vacuum-sealed chocolates were then stored inside a freezing chamber at −20 °C. Prior to tasting, the chocolates were allowed to defreeze at room temperature for two days.

**Sensory Analysis**—All tasting sessions were carried out at CIRAD’s (Montpellier, France) sensory analysis laboratory, in individual boxes and under a red light. Blind sensory analysis was performed on 54 dark chocolate samples by a panel composed of 13 trained tasters, 6 of which were female and 7 were male, aged between 21 and 60 years (all members of CIRAD’s internal sensory panel).

The 13 panelists, who were already familiar with dark chocolate sensory evaluation, were trained throughout six training sessions for the purpose of this analysis. Six different dark chocolates were used for this training, each exhibiting distinctive and intense attributes in particular (e.g., cocoa, bitter, yellow fruits, etc.). The chocolates used for the training were either provided by Barry Callebaut AG and CIRAD, or they were bought from local chocolatiers in France. During the first two training sessions, open discussions were held on the perceived attributes of each of the chocolates, In the course of the next four sessions, the chocolates were then blindly tasted, repeatedly. The performance of the panelists was validated based on their repeatability and agreement with the rest of the panel.

Eight sessions distributed over six weeks were needed for the tasting and evaluation of the 54 chocolates. In each session, six chocolates were evaluated, one of which was a replicate sample taken randomly within that session’s set of samples. The sensory attributes (sweetness, bitterness, acidity, astringency, cocoa, fruity-yellow fruits, fruity-red fruits, fruity-citrus, fruity-dried fruits, nutty, winey, black olives, green, earthy, floral, woody, spicy, and roasted) were evaluated using a score that ranged from 0 to 10. For each attribute, the *mean sensory score* was calculated from the scores given by the eight most discriminant panelists. The most discriminant panelists were those who gave a wider range of scores to each attribute among different samples, effectively demonstrating their ability to best differentiate the subtleties between chocolate samples.

**Volatile Analysis**—Dark chocolate samples were frozen in liquid nitrogen before being milled using a conventional coffee mill. The retrieved powder was then sieved and stored in a freezer at −20 °C. Volatile compounds were extracted from 2 g of sieved sample powder by means of headspace solid-phase micro extraction (SPME-HS), using a 50/30-μm divinylbenzene/carboxene/polydimethylsiloxane (DVB/CAR/PDMS) fiber (Supelco Analytical Products—Sigma Aldrich, Merck, Darmstadt, Germany). The extracted volatile compounds were analyzed using an Agilent 6890 N gas chromatography–mass spectrometer (GC–MS) equipped with a capillary column DBWAX, 60 m length × 0.25 mm internal diameter × 0.25 μm film thickness (Agilent, Santa Clara, California, USA). The full procedure for volatile compound extraction and identification was previously described by Assi-Clair et. al. [57]. The relative concentration of each compound was calculated based on the exact weight of the sample and on the known concentration of the compound that was added as an internal standard (butan-1-ol). Each sample was analyzed in triplicate and the mean of the three obtained concentrations per sample was recovered for reporting (in μg/g of fresh matter) and for the subsequent statistical analysis.

**Statistical Data Analysis**—Statistical analysis, including chemometric analysis based on the principal component analysis (PCA) and on partial least squares (PLS) regression, was performed with XLSTAT STUDENT (Addinsoft, Paris, France).

PLS descriptive and predictive models were built in R, using the ‘caret’ package. All variables were centered and scaled. The dataset was split into training and testing sub-datasets. PLS training models were built, using the ‘trainControl()’ function. The training models were optimized by cross-validation following the “repeatedcv” method, by repeatedly partitioning the data into a fixed number of equally sized groups (20 folds were used) and then training and testing the model using each group as the validation set. This process was then repeated 30 times. The metric used in the cross-validation with the training data subset was the receiver operating characteristic (ROC).

The best models obtained from the training data subset were then validated using the testing data subsets. This operation was repeated in a loop in order to cover the entirety of the dataset, iteratively training and testing the PLS models on different subsets of the data. The ‘predict()’ function was used to obtain predictions for the test set based on the trained models for each attribute. In order to determine the best fitted predictive model per attribute, the following performance indicators were taken into consideration: accuracy, confidence interval, sensitivity, and specificity.

## 5. Conclusions

The results of this study provide new insights into the link between the volatile composition and the perceived flavor of dark chocolates processed from cocoa beans sourced from the Dominican Republic. This study helped identify certain volatile compounds that are important in predicting the intensity of the sensory attributes of interest in dark chocolate samples. Acetaldehyde, dimethyl sulfide, and 2,3-butanediol were found to be key predictors in identifying the intensity at which multiple sensory attributes may be perceived, including bitterness, astringency, citrus, acidity, red fruits, dried fruits, green, black olives, woody, and sweetness. While the relative concentrations of these compounds seem to be key in describing and predicting the flavor intensity of multiple attributes, propylene glycol diacetate was identified as a unique key compound in describing and predicting a single attribute (red fruit aroma).

This study has also demonstrated the potential to differentiate chocolates with relative accuracy based on their flavor profiles, using predictive models based solely on the volatile composition of the analyzed chocolates. The efficacy of these models could be improved by incorporating more samples, leading to more accurate flavor profile predictions. Furthermore, these models could potentially be used by industry players as a reliable, repeatable, and inexpensive tool, which would spare them the costs of using human sensory panels for the classification and evaluation of chocolate and other cocoa products. As such, the results of this study open up new avenues for the cocoa and chocolate industries to evaluate and optimize their flavor quality.

## Figures and Tables

**Table 1:** Volatile compounds identified in the 54 dark chocolate samples, along with their associated flavor descriptors.

Compound Group	Compound Name	Odor/Flavor Attributes
Aldehydes	2-methylbutanal	Chocolate [35]
	2-methylpropanal	Chocolate [35]
	2-phenylacetaldehyde	Berry, geranium, honey [36]
	3-methylbutanal	Chocolate [35]
	Acetaldehyde	Tart (acidic), pungent fruity [36]
	Benzaldehyde	Nutty, almond [35]
Esters	1,3-diacetoxypropane	Acetic, fruit [37]
	Diethyl butanedioate	Cotton, fabric, floral, fruit [36]
	Ethyl acetate	Pineapple [14]
	Isoamyl acetate	Fruity, banana [36]
	Propylene glycol diacetate	Fruit [36]
Alcohols and Phenols	3-methylbutan-1-ol	Pungent, repulsive taste [36]
	2,3-butanediol (isomere A)	Sweet [36]
	2,3-butanediol (isomere B)	Sweet [36]
	2-methylpropan-1-ol	Sweet, whiskey [36]
	2-phenylethanol	Rose, lilac, flowery, caramel [28]
	Acetoin	Buttery [36]
	Ethanol	-
	Furfuryl alcohol	Bitter [36]
	Pentan-2-ol	Fuel oil, green [36]
Ketones	Acetoin acetate	Fruit [36]
	Acetol	Pungent, sweet, caramel-like [36]
	Acetophenone	Must, flower, almond, sweet [38]
	Butane-2,3-dione	Buttery [36]
Pyrazines	2,3,5,6-tetramethylpyrazine	Milk-coffee, roasted, chocolate [35]
	2,3,5-trimethylpyrazine	Cocoa, roasted, cooked [35]
	2,3-dimethylpyrazine	Cooked, nutty [35]
Other	2,2,4,6,6-pentamethylheptane	Unspecified
	2-acetylpyrrole	Bread, cocoa, hazelnut, licorice, walnut [36]
	Acetic acid	Sour, astringent, vinegar [35]
	Butyrolactone	Sweet, caramel-like [36]
	Decane	Gasoline-like [36]
	Dimethyl sulfide	Unpleasant, cabbage-like [36]
	Toluene	Fuel-like [36]
	Unidentified compound	Unspecified

**Table 2:** Training (estimated by cross-validation) and validation (tested in-loop, with the whole data set) test results of the best fitted models obtained for predicting samples classified as “above median”.

	Training Dataset Metrics				Validated Model Performance for Prediction			
Attribute Model	Number of Components	ROC	Sensitivity	Specificity	Accuracy	95% CI	Sensitivity	Specificity
Bitterness	4	0.8508	0.8117	0.7300	0.7963	(0.6647, 0.8937)	0.7714	0.8421
Astringency	3	0.9263	0.8083	0.8608	0.7963	(0.6647, 0.8937)	0.8214	0.7692
Citrus	1	0.8146	0.6600	0.7283	0.7222	(0.5836, 0.8354)	0.7692	0.6786
Acidity	3	0.7621	0.6817	0.7008	0.7037	(0.5639, 0.8202)	0.7500	0.6667
Red fruits	3	0.8579	0.7058	0.7867	0.7037	(0.5639, 0.8202)	0.7407	0.6667
Dried fruits	2	0.7846	0.6283	0.8250	0.6852	(0.5445, 0.8048)	0.7273	0.6562
Green	2	0.8192	0.8225	0.6792	0.6852	(0.5445, 0.8048)	0.7143	0.6316
Black olives	1	0.6538	0.7342	0.5933	0.6667	(0.5253, 0.7891)	0.6562	0.6818
Woody	4	0.8038	0.7550	0.7500	0.6667	(0.5253, 0.7891)	0.6071	0.7308
Sweetness	3	0.7767	0.7042	0.7550	0.6667	(0.5253, 0.7891)	0.6667	0.6667

**Table 3:** Compilation of the five variables with the highest relative importance in building the above-median-models, together with their variable importance in projection (VIP) and Pearson correlation coefficient.

Attribute Model	Variable	VIP	Corr. Coeff.	Attribute Model	Variable	VIP	Corr. Coeff.
Bitterness	Acetaldehyde	100.00	−0.782	Dried fruits	Ethyl Acetate	100.00	−0.554
	Dimethyl sulfide	64.98	0.594		3-methylbutan-1-ol	96.36	−0.566
	2,3-butanediol (isomere A)	49.53	−0.694		Ethanol	91.13	−0.439
	3-methylbutan-1-ol	41.43	0.598		Acetaldehyde	89.82	0.505
	Acetic acid	37.21	−0.636		Pentan-2-ol	87.1	−0.553
Astringency	Acetaldehyde	100.00	−0.749	Green	Acetaldehyde	100.00	−0.571
	3-methylbutanal	79.16	−0.632		Diethyl butanedioate	86.36	0.344
	2,3-butanediol (isomere A)	75.10	−0.622		Dimethyl sulfide	82.36	0.529
	2-methylpropanal	72.22	−0.487		3-methylbutanal	77.51	−0.589
	Dimethyl sulfide	59.42	0.531		Butane-2,3-dione	60.84	−0.411
Citrus	2,3-butanediol (isomere B)	100.00	0.596	Black olives	Dimethyl sulfide	100.00	0.530
	Acetic acid	96.23	0.596		2,3-butanediol (isomere A)	92.87	−0.591
	3-methylbutan-1-ol	91.39	−0.564		Acetic acid	88.45	−0.486
	Ethyl Acetate	88.51	−0.503		Pentan-2-ol	75.66	0.310
	Pentan-2-ol	87.34	−0.532		2,3-butanediol (isomere B)	71.44	−0.340
Acidity	Acetaldehyde	100.00	0.603	Woody	3-acetyloxypropyl acetate	100.00	0.151
	Butyrolactone	73.30	0.451		Unknown compound	98.14	0.248
	Isoamyl acetate	65.34	0.179		Acetaldehyde	91.19	−0.561
	2,3-butanediol (isomere A)	64.38	0.557		2,3-butanediol (isomere A)	84.28	−0.626
	2,3-butanediol (isomere B)	64.24	0.567		Butyrolactone	78.3	−0.257
Red fruits	Acetaldehyde	100.00	0.628	Sweetness	Acetaldehyde	100.00	0.605
	Propylene glycol diacetate	83.08	0.665		Acetol	89.51	−0.042
	2,3-butanediol (isomere A)	57.04	0.607		Dimethyl sulfide	73.49	−0.557
	Dimethyl sulfide	51.26	−0.506		2,3-butanediol (isomere A)	67.46	0.582
	2,3-butanediol (isomere B)	47.27	0.534		3-methylbutanal	59.84	0.512

**Table 4:** Validation test results of the models obtained for predicting “high” sensory scores for bitterness and astringency, along with the five most important variables, their VIP, and Pearson correlation coefficients.

	Looped Tested Model Performance						
Attribute Model	Accuracy	95% CI	Sensitivity	Specificity	Variable	VIP	Corr. Coeff.
Bitterness	0.7963	(0.6647, 0.8937)	0.8919	0.5882	Acetaldehyde	100.00	−0.782
					2,3-butanediol (isomere A)	67.29	−0.694
					3-methylbutan-1-ol	65.25	0.598
					2,3-butanediol (isomere B)	62.41	−0.548
					Dimethyl sulfide	57.28	0.594
Astringency	0.8148	(0.6857, 0.9075)	0.8974	0.6000	2,3-butanediol (isomere A)	100.00	−0.622
					Acetaldehyde	98.52	−0.749
					Acetic acid	97.44	−0.519
					2,3-butanediol (isomere B)	88.46	−0.378
					3-methylbutan-1-ol	86.76	0.397

## Data Availability

The data presented in this study are not available.

## Sample Availability

No sample is available from the authors.

## Appendix A

**Table A1:** Average, standard deviation, minimum, and maximum values of the mean sensory scores of all samples per sensory attribute evaluated. The scores ranged from 0 to 10.

Sensory Attribute	MEAN	STDEV	MAX	MIN
Sweetness	4.245	0.535	5.125	3.125
Bitterness	4.108	0.991	6.750	2.750
Acidity	3.997	0.686	6.000	2.375
Astringency	4.038	0.762	5.375	2.250
Cocoa	5.447	0.529	6.563	4.000
Yellow fruits	2.598	0.983	4.750	0.750
Red fruits	2.703	1.033	5.125	0.500
Citrus	2.288	0.866	4.250	0.375
Dried fruits	2.089	0.710	3.625	0.500
Nutty	2.082	0.585	3.625	1.000
Winey	1.258	0.492	2.375	0.250
Black olives	1.050	0.506	2.500	0.125
Green	1.274	0.629	2.875	0.375
Earthy	1.627	0.625	3.375	0.625
Floral	2.232	0.739	4.000	0.625
Woody	1.525	0.525	2.750	0.625
Spicy	2.729	0.635	4.375	1.500
Roasted	2.533	0.519	3.750	1.625

**Table A2:** Average, standard deviation, minimum, and maximum values of the relative concentrations of all identified volatile compounds in the entire sample dataset. The relative concentration was reported in μg/g of fresh matter.

Volatile Compound	MEAN	STDEV	MAX	MIN
Acetaldehyde	0.341	0.099	0.550	0.146
Dimethyl sulfide	0.092	0.024	0.145	0.048
2-methylpropanal	0.319	0.074	0.489	0.166
Ethyl acetate	29.288	19.128	82.080	6.820
2-methylbutanal	0.467	0.136	0.838	0.255
3-methylbutanal	1.454	0.369	2.437	0.927
Ethanol	3.032	1.940	9.798	0.499
2,2,4,6,6-pentamethylheptane	20.881	7.421	41.752	7.318
Butane-2,3-dione	5.061	1.038	7.746	3.044
Decane	0.229	0.140	0.602	0.017
Toluene	0.794	0.763	2.900	0.030
Isoamyl acetate	1.325	0.825	6.224	0.558
2-methylpropan-1-ol	1.304	0.824	6.300	0.605
Pentan-2-ol	2.081	1.098	5.184	0.805
3-methylbutan-1-ol	0.745	0.394	1.872	0.213
Acetoin	15.747	3.984	25.335	8.412
Acetol	7.988	2.160	13.707	4.305
2,3-dimethylpyrazine	0.687	0.356	1.797	0.178
Acetoin acetate	2.830	1.063	5.550	1.183
2,3,5-trimethylpyrazine	2.688	1.229	5.724	0.857
Acetic acid	297.584	67.181	416.201	177.745
2,3,5,6-tetramethylpyrazine	27.517	17.194	92.471	5.735
Benzaldehyde	1.767	0.797	3.913	0.595
Propylene glycol diacetate	1.309	0.950	4.243	0.192
2,3-butanediol (isomere A)	37.814	10.784	55.346	17.717
2,3-butanediol (isomere B)	12.679	4.402	21.477	5.786
Butyrolactone	1.456	0.214	2.112	1.115
2-phenylacetaldehyde	0.568	0.357	1.909	0.145
Acetophenone	0.500	0.242	1.292	0.155
1,3-Diacetoxypropane	4.828	7.718	34.468	0.098
Furfuryl alcohol	0.166	0.038	0.248	0.108
Diethyl butanedioate	0.156	0.153	0.714	0.004
Unidentified Compound	10.470	15.051	64.091	0.331
2-phenylethanol	4.958	2.097	10.207	1.677
2-Acetylpyrrole	0.451	0.209	1.033	0.165
